# KCNE1 rs1805127 Polymorphism Increases the Risk of Atrial Fibrillation: A Meta-Analysis of 10 Studies

**DOI:** 10.1371/journal.pone.0068690

**Published:** 2013-07-18

**Authors:** Chang Liang, Xiankai Li, Yawei Xu, Qingyong Chen, Yadong Wu, Wan Wang, Weiming Li, Mantang Qiu

**Affiliations:** 1 Department of Cardiology, Shanghai Tenth People's Hospital, Tongji University, Shanghai, China; 2 The Key Laboratory of Cardiovascular Disease and Molecular Intervention of Nanjing Medical University, Nanjing, China; 3 The Fourth Clinical College of Nanjing Medical University, Nanjing, China; 4 Department of Thoracic Surgery, Cancer Institute of Jiangsu Province, Nanjing, China; Loyola University Chicago, United States of America

## Abstract

**Background:**

Atrial fibrillation (AF) is one of the most common types of arrhythmia in humans. Recently, many studies have investigated the relationship between human atrial fibrillation and the single nucleotide polymorphism (SNP) of rs1805127 (A>G) in KCNE1 gene, but the results were still inconsistent and inconclusive.

**Method:**

Electronic databases and bibliographies of retrieved studies were searched. We performed a meta-analysis of ten case-control studies, including 2099 cases and 2252 controls, to evaluate the association of rs1805127 polymorphism (A>G) with the risk of AF. Random-effects model was used when the heterogeneity was obvious; otherwise, fixed-effects model was applied. Meta-regression was performed to examine potential source of heterogeneity. Egger's test and Begg's test were used to detect publication biases.

**Results:**

The results showed a significantly increased risk of AF in homozygote comparison (GG vs. AA:OR = 1.899, 95%CI: 1.568, 2.300; P_heterogeneity_ = 0.217), heterozygote comparison (GA vs. AA:OR = 1.436, 95% CI:1.190, 1.732; P_heterogeneity_ = 0.739), dominant model(GA /GG vs. AA: OR = 1.624, 95%CI: 1.361, 1.938; P_heterogeneity_ = 0.778) and recessive model (GG vs. GA/AA: OR = 1.394, 95%CI:1.152, 1.686; P_heterogeneity_ = 0.03). Meta-regression revealed that the sample size and the types of AF were the source of the heterogeneity.

**Conclusion:**

The rs1805127 polymorphism (A>G) of KCNE1 is associated with an increased risk of AF, which suggests the rs1805217 polymorphism of KCNE1 gene may play an important role in the pathogenesis of AF.

## Introduction

Atrial fibrillation (AF) is the most common type of sustained tachyarrhythmia in human, and the prevalence increased year by year. However, the pathogenesis of AF remains unclear. Currently, a number of studies have shown that mutations in ion channel genes may be the risk factors of AF [1], [2].

The KCNE1 gene was first discovered by Murai in 1989[3], which located in 21q22.1-22.2. KCNE1 encodes the β-subunits of the delayed rectifier potassium current channel (IKs) in human heart, which is also called Mink protein, including 130 amino acids. The functional rs1805127 polymorphism (A>G) of KCNE1 gene leads to a serine to glycine substitution [4]. Over the last decade, a number of case-control studies have been conducted to investigate the association between rs1805127 polymorphism and AF. Lai reported that the variant G allele was more common in AF patients compared with that of control subjects, the result showed that KCNE1 polymorphism was associated with atrial fibrillation [5]. This conclusion was supported by the studies reported by Prystupa [6] and Fatini [7]. However, this association was not observed in other studies [8]–[11]. During the past two years, three case-control studies [12]–[14] with larger sample size were performed, but the result remain inconsistent and inconclusive.

To determine the association between KCNE1 rs1805127 polymorphism and the risk of AF, we performed this meta-analysis of published case-control studies to attain better results and hence more details and accurate risk estimation.

## Methods

We done this meta-analysis according to the PRISMA guideline (Supporting information: Table S1). Appropriate case-control studies were extracted by electronic search of databases and manual search of references of relative articles and reviews. We searched PubMed/MEDLINE, EMBASE, and China National Knowledge Infrastructure (CNKI) using key words of “KCNE1 or potassium voltage-gated channel, Iks-related family, member 1”,“polymorphism or variant” and “atrial fibrillation or AF”. There were no limitation of research data and the last research was performed on 8 January, 2012. References of related studies and reviews were manually searched for additional studies.

### Inclusion and Exclusion Criteria

Inclusion criteria were as follows: (1) case-control studies; (2) investigating the association between the KCNE1 rs1805127 (A>G) polymorphism and risk of AF; (3) studies that provide detailed genotypes data (AA, GA and GG) in AF cases and control groups. Exclusion criteria were as follows: (1) duplicated studies; (2) no controls; (3) no detail genotype frequencies. We did not consider unpublished reports, abstracts, comments, reviews, or editorials. Two reviewers extracted eligible studies independently according to the inclusion criteria. Disagreements between two reviewers were discussed with another reviewer till consensus was achieved.

### Data Extraction

Data extraction was conducted independently by two authors with disagreements resolved by discussion. The following data were extracted from eligible study: the first authors' name, publication year, country where the study was conducted, genotyping methods, ethnicity, source of control, Hardy-Weinberg equilibrium (HWE), number of cases and controls, genotype frequency in cases and controls. Different ethnicities were divided into Asian and Caucasian. Source of control in the studies were defined as hospital-based (HB) and population-based (PB). An online program (http://ihg.gsf.de/cgi-bin/hw/hwa1.p1) was used to test the Hardy-Weinberg equilibrium in the controls [15], and a p<0.05 indicates disequilibrium of HWE.

### Statistical Analysis

Odds ratios (OR) and 95% confidence intervals (CI) were used to measure the association strength between the KCNE1 rs1805127 polymorphism and AF risk. In this meta-analysis, we evaluated the overall AF risk in four comparison models: homozygote comparison (GG vs. AA), homozygote comparison (GA vs. AA), recessive model (GG vs. GA/AA) and dominant model (GG/GA vs. AA). Subgroup analyses were carried out to explore the confounding factors: ethnicities, source of control and sample size. Sensitivity analyses were performed to identify individual study effect on pooled results and test the reliability of results.

The Cochran's Q statistic was used to evaluate the heterogeneity between studies [16]. When the P value was <0.10, the heterogeneity were considered as significant, and the pooled AF risk was estimated by the random-effects model; otherwise, fixed-effects model was applied [17]. Meta-regression analysis was performed to find the source of heterogeneity, and a p<0.05 was considered significant [18].

We used visual inspection of asymmetry in funnel plots, Begg's test and Egger's test to statistically potential publication bias, and a P<0.05 was considered significant [19]. All statistical analyses were calculated with STATA software (StataCorp, College Station, TX). And all P values were two-side with a significance level of 0.05.

## Results

### Characteristics of eligible studies

**Figure 1:**
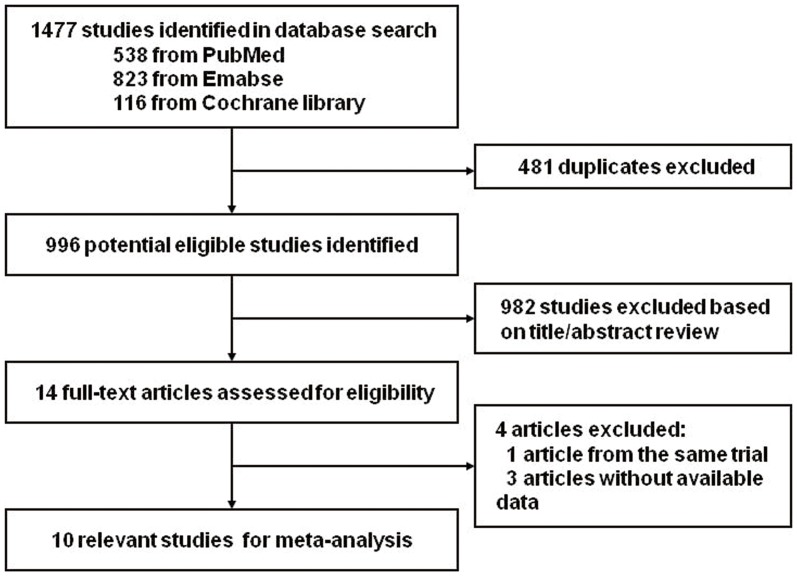
PRISMA Flow Chart. A total of 14 articles were identified and articles from the same trial and 3 articles without available data, thus 10 studies were suitable.

Finally, 10 studies investigating the association between the rs1805127 polymorphism of KCNE1 gene and AF were identified according to the inclusion and exclusion criteria, including 2099 cases and 2252 controls [4]–[6], [8]–[14]. The detailed screening process was showed in Figure 1, and the characteristics of eligible studies are listed in Table 1. Blood sample was used for genotyping in all studies. Among the ten studies, eight were performed in Asian; two were performed in Caucasian. The sample size of four of the ten is larger than 300 (classified as “large”), and six of the ten were less than 300 (classified as small). Most of the studied are Hospital-Based (HB, seven studies), and the others were Population-Based (PB). HWE of genotype distribution in the controls was tested by the online program and the genotypes distribution in controls was not in agreement with HWE in two studies [8], [9]. Since no genotype error was detected, these two studies were not excluded.

**Table 1:** Eligible Studies and data.

first author	year	country	ethnicity	source of control	Cases			Controls		
					GG	GA	AA	GG	GA	AA
Lingping Lai [9]	2002	China	Asian	HB	64	37	7	46	44	18
Ni Aizhen [10]	2004	China	Asian	PB	54	37	3	72	48	10
Cinzia Fatini [5]	2006	Italy	Caucasian	HB	118	155	58	116	207	118
Andrzej prytupa [6]	2006	Poland	Caucasian	PB	24	38	7	3	45	13
LOU Sheng [7]	2006	China	Asian	PB	63	41	7	60	29	12
ZENG Zhi-yu [11]	2007	China	Asian	PB	71	60	10	55	54	11
Xu Lixin [8]	2008	China	Asian	HB	77	61	9	75	56	16
Yao Juan [15]	2011	China	Asian	HB	158	117	28	129	159	40
Yao Juan [16]	2012	China	Asian	HB	133	138	36	118	148	64
Mao Ting [17]	2012	China	Asian	HB	218	201	69	188	215	85

PB: population-based; HB: hospital-based.

### Result of meta-analysis

**Figure 2:**
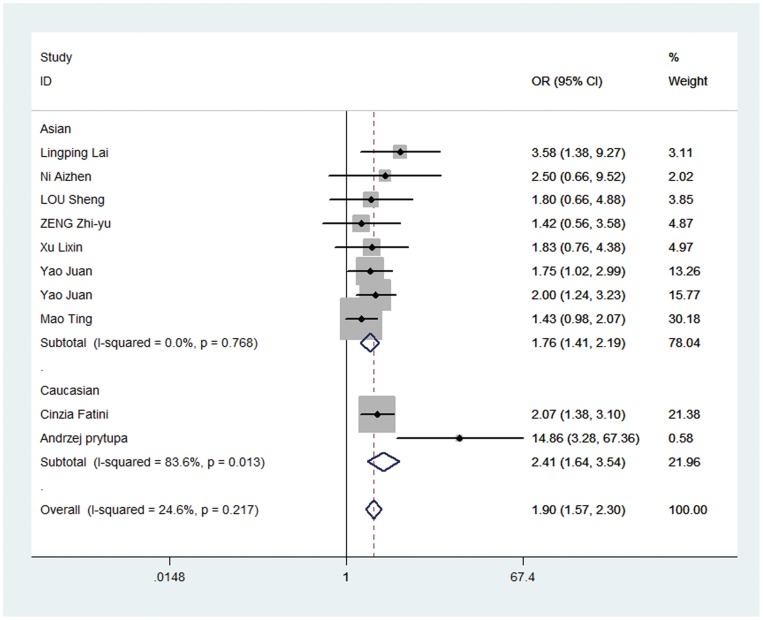
Forest plot of homozygous comparison (GG vs. AA) for overall comparison in fixed-effects model. (OR = 1.899, 95%CI: 1.568, 2.300; P_heterogeneity_ = 0.217).

**Figure 3:**
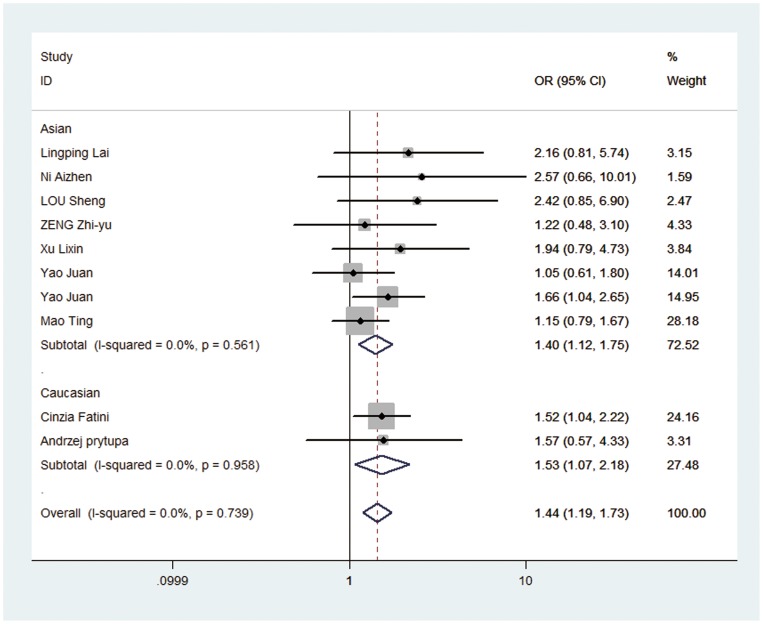
Forest plot of heterozygous comparison (GA vs. AA) for overall comparison in fixed-effects model. (OR = 1.436, 95%CI: 1.190, 1.732; P_heterogeneity_ = 0.739).

**Figure 4:**
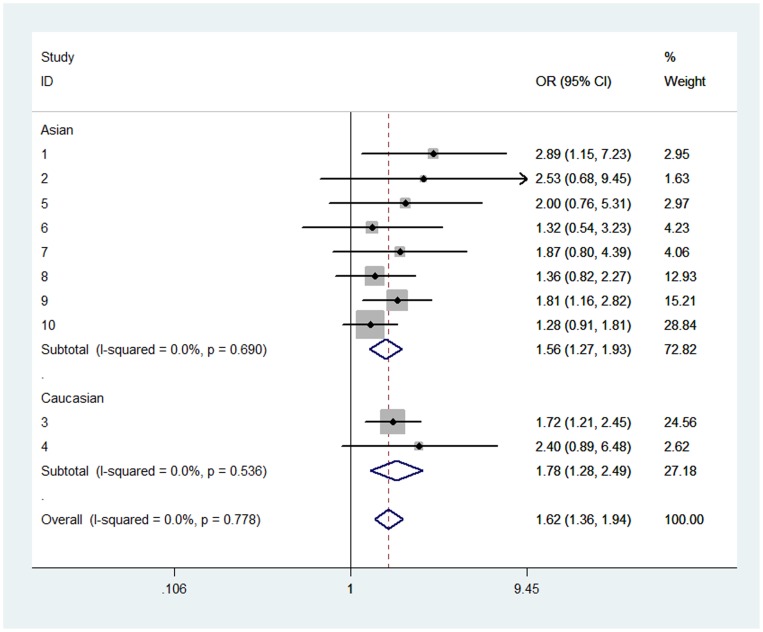
Forest plot of dominant model (GA/GG vs. AA) for overall comparison in fixed-effects model. (OR = 1.624, 95%CI: 1.361, 1.938; P_heterogeneity_ = 0.778).

**Figure 5:**
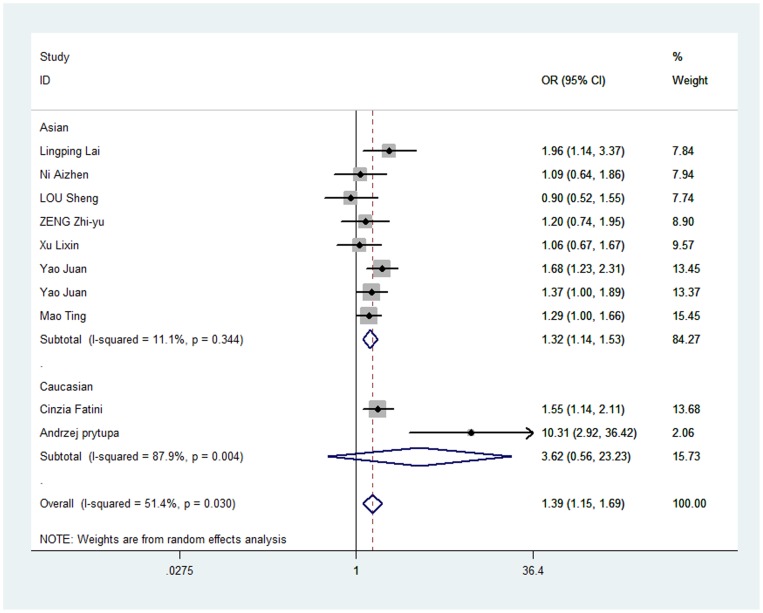
Forest plot of recessive model (GG vs. GA/AA) for overall comparison in random-effects model. (OR = 1.394, 95%CI: 1.152, 1.686; P_heterogeneity_ = 0.03).

In overall analysis, we found rs1805127 polymorphism was associated with increased risk of AF in all four comparisons: homozygote comparison (GG vs. AA: OR = 1.899, 95%CI: 1.568, 2.300; P_heterogeneity_ = 0.217, Table 2, Figure 2), heterozygote comparison (GA vs. AA: OR = 1.436, 95%CI: 1.190, 1.732; P_heterogeneity_ = 0.739, Table 2, Figure 3), dominant model (GA/GG vs. AA: OR = 1.624, 95%CI: 1.361, 1.938; P_heterogeneity_ = 0.778, Table 2, Figure 4) and recessive model (GG vs. GA/AA: OR = 1.394, 95%CI: 1.152, 1.686; P_heterogeneity_ = 0.03, Table 2, Figure 5).

**Table 2:** Meta-analysis results.

		GG vs. AA		GA vs. AA		GA/GG vs. AA		GG vs. GA/AA	
	N	OR	P_h_	OR	P_h_	OR	P_h_	OR	P_h_
Total	10	1.899(1.568,2.300)*	0.217	1.436(1.190,1.732)*	0.739	1.624(1.361,1.938)*	0.778	1.394(1.152,1.686) *	0.03
Ethnicities									
Asian	8	1.756(1.407,2.191) *	0.768	1.401(1.122,1.748)*	0.561	1.564(1.270,1.926)*	0.690	1.328(1.160,1.521)*	0.344
Caucasian	2	4.822(0.711,32.679)	0.107	1.529(1.074,2.177)*	0.958	1.785(1.280,2.490)*	0.536	3.615(0.563,23.228)	0.004
Source of control									
HB	7	1.831(1.491,2.248)*	0.532	1.393(1.140,1.703)*	0.535	1.586(1.314,1.916)*	0.531	1.433(1.250,1.641) *	0.419
PB	3	2.724(1.111,6.679)*	0.067	1.757(1.040,2.967)*	0.731	1.917(1.160,3.167)*	0.792	1.482(0.779,2.820)	0.006
Sample size									
Large study	4	1.764(1.420,2.192)*	0.145	1.338(1.083,1.653) *	0.919	1.525(1.251,1.859)*	0.889	1.445(1.246,1.675)*	0.581
Small study	6	2.460(1.628,3.719)*	0.552	1.862(1.236,2.805)*	0.447	2.062(1.396,3.047)*	0.534	1.404(0.927,2.127)	0.008

num: number of studies included; OR: odds ratio; P_h_: p value for heterogeneity; PB: population-based; HB: hospital-based; *OR with statistical significance; large study: studies with more than 300 participants; small study: studies with less than 300 participants.

Subgroup analyses for ethnicity suggested that the rs1805127 polymorphism was associated with an increased risk of AF among Asian population (Table 2). But for Caucasian population, the increased risk of AF was observed in heterozygote comparison and dominant model, but not in homozygous comparison and recessive model (Table 2) Analyses by source of control showed that there was significant association between the rs1805127 polymorphism of KCNE1 and increased risk of AF both in HB and PB studies (Table 2). Stratified analyses by sample size also suggested that the rs1805127 polymorphism (A>G) of KCNE1 increased risk of AF both in large studies and small studies (Table 2).

### Heterogeneity and Sensitivity Analysis

The result of heterogeneity between included studies were shown in Table 2. Heterogeneity was significant in recessive model of overall analysis. We performed meta-regression to detect source of heterogeneity and sensitivity analysis to explore each study's influence on the total results. Meta-regression revealed that source of control (P = 0.047) and ethnicity (P = 0.035) but not sample size (P = 0.337) were the sources of heterogeneity, which was inconsistent with sub-group analyses results (Table 2). Sensitivity analysis suggested that the study published by Prytupa [6] was responsible for the heterogeneity. After removing this study, the results did not change significantly (OR = 1.362, 95%CI: 1.203, 1.541), and the no significant heterogeneity was observed (P = 0.369).

### Test for Potential Publication Bias

**Figure 6:**
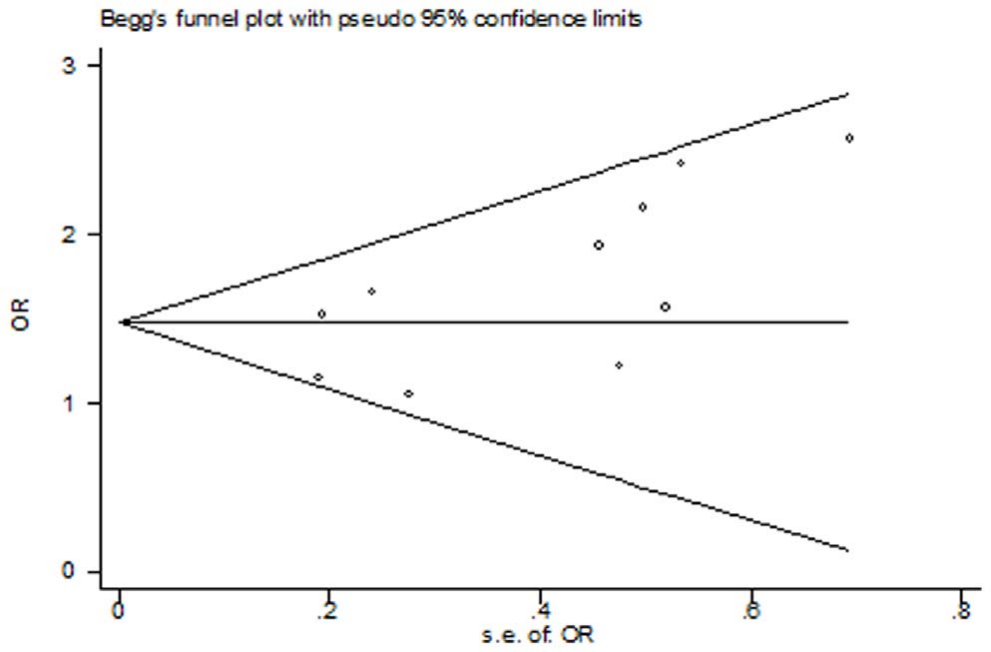
Funnel plot of heterozygote comparison (GA vs. AA) in all 10 eligible studies, Begg's test p = 0.049.

**Figure 7:**
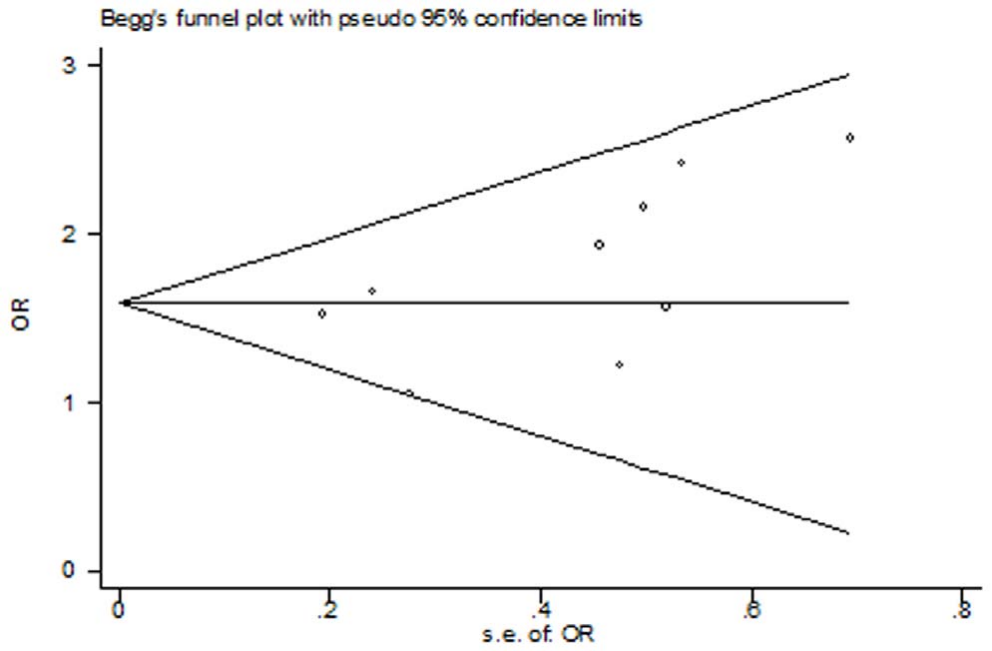
Funnel plot of heterozygote comparison (GA vs. AA) in 9 studies (Mao' study was excluded), Begg's test p = 0.128.

We assessed publication bias by Begg's funnel plot and Egger's test. The results showed no evidence of publication bias (homozygote comparison: P_Begg_ = 0.097; dominant model: P_Begg_ = 0.052, recessive model GG vs. GA/AA: P_Begg_ = 0.084), except for heterozygote comparison (GA vs. AA: P_Begg_ = 0.049, Figure 6), further study suggest that the study of Mao [14] was responsible for the asymmetry of funnel plot; we performed trim and fill method. When this title was deleted, there was no evidence of publication bias (GA vs. AA: P_Begg_ = 0.128, Figure 7), and the pooled OR was still significant (OR = 1.547, 95% CI: 1.244, 1.924, P_heterogeneity_ = 0.834).

## Discussion

KCNE1 gene encodes the β-subunits of the delayed rectifier potassium current channel (IKs) in human heart. The delayed rectifier potassium current channel (IKs) is important for cardiac repolarization, especially at the late phase of phase 3 of action potential, and it's closely related to action potential duration [20]–[22]. Recently, many studies have investigated the association between IKs function and heart arrhythmia, mostly in long Q-T syndrome and AF [21], [23]–[26]. Ehrlich and colleagues demonstrated that the rs1805127 G-allele carriers have a higher prevalence of tachyarrhythmia resulting from decreased IKs current, prolonged action potential duration, prolonged relative refractory period, inducing early afterdepolarization under specific conditions [19]. The study reported by Chevillard confirmed that the KCNE1 gene was over-expressed in atrial tissue in patients with permanent atrial fibrillation [24]. It was also found that the protein KCNE1 gene encoded may work through interacting with other proteins, forming arrhythmogenic substrate and resulting in atrial fibrillation and maintaining [21]. These results show that KCNE1 gene is important in the regulation of heart rhythm.

In this meta-analysis, we analyzed 10 studies including 2099 cases and 2252 controls and found a significant association between the rs1805127 polymorphism (A>G) of KCNE1 and increased risk of AF in all comparison models of overall analysis.

In subgroup analyses of ethnicity, the significant association was observed in all comparisons in Asian population, but not all comparisons in Caucasian. Small sample size and limited number of studies of Caucasian in this meta-analysis (only two studies available [6], [7]) maybe the major reasons of these discrepancies. Additionally, the heterogeneity between two studies was significant (Table 2), which may be also contributed by limited number of studies. The different AF risks in Asians and Caucasians was also reported in other meta-analysis [27]. Additional studies with lager sample sizes will be necessary to clarify this finding.

Subgroup analysis of source of control also showed a positive association, except the recessive model, in PB studies. However, studies of PB were not all complied with HWE[4], [9], and other heart disease may be an incentive of AF, like coronary heart disease and valvular heart disease, thus, studies of PB control could not match all high risk factors of AF between case group and control group. Consequently, setting HB persons as a control group is more representative for the result and encounters less bias, which matched cases and controls according to various risk factors. Since both results from PB and HB suggested that the rs1805127 polymorphism increased risk of AF, this conclusion is reliable.

For heterogeneity, in overall analysis, the results showed no significant heterogeneity except in recessive model. Indicated by the results of sensitivity analysis of recessive model, the study by Prytupa [6] was considered as the source of heterogeneity. Firstly, sample size of Prytupa's study was small, with only 69 cases and 60 controls. Secondly, the case group of Prytupa's study was based on lone paroxysmal AF and patients with permanent AF were excluded, which was different from other studies. However, this kind of heterogeneity was difficult to avoid because enough sample of kinds of AF types was difficult. Additionally, meta-regression revealed that source of control and ethnicity but not sample size contributed to heterogeneity.

In this meta-analysis, we analyzed all eligible studies about the association between rs1805127 polymorphism (A>G) of KCNE1 gene and risk of AF, including 2099 cases and 2252 controls. There was no limitation of languages when searching, and the results showed a low chance of publication bias. Some limitation of our meta-analysis should be considered. Firstly, AF was considered as a multi-factorial disease, to achieve more precise estimate, individual data should be adjusted according to some other variables that related to risk of AF, such as family history and coronary heart disease. Secondly, as reported by Fatini [7] and Xu [10], whether there was a joint interaction between rs1805127 polymorphism of KCNE1 and polymorphisms of other genes (such as eNOS T786C gene polymorphism) in the development of AF is unknown. Further experiment studies are needed to find the mechanism of the combined effects. Thirdly, the AF types were not fully specified in studies, uniform adjustment is needed for the diagnosis of AF in each study.

In conclusion, our meta-analysis demonstrates that the rs1805127 polymorphism (A>G) of KCNE1 gene is associated with increased risk of AF; this finding suggests that the rs1805217 polymorphism of KCNE1 gene may play an important role in the pathogenesis of AF. To confirm this association, further experiment studies are warranted.

## Supporting Information

Table S1 **PRISMA checklist.**
(DOC)
